# Terpenoids and Polyphenols as Natural Antioxidant Agents in Food Preservation

**DOI:** 10.3390/antiox10081264

**Published:** 2021-08-08

**Authors:** Ignacio Gutiérrez-del-Río, Sara López-Ibáñez, Patricia Magadán-Corpas, Luis Fernández-Calleja, Álvaro Pérez-Valero, Mateo Tuñón-Granda, Elisa M. Miguélez, Claudio J. Villar, Felipe Lombó

**Affiliations:** 1 Research Group BIONUC (Biotechnology of Nutraceuticals and Bioactive Compounds), Departamento de Biología Funcional, Área de Microbiología, Universidad de Oviedo, 33006 Oviedo, Spain; UO217717@uniovi.es (I.G.-d.-R.); saralopez@uvigo.es (S.L.-I.); UO166365@uniovi.es (P.M.-C.); UO238083@uniovi.es (L.F.-C.); UO279586@uniovi.es (Á.P.-V.); UO252176@uniovi.es (M.T.-G.); emmiguelez@uniovi.es (E.M.M.); cjvg@uniovi.es (C.J.V.); 2 IUOPA (Instituto Universitario de Oncología del Principado de Asturias), 33006 Oviedo, Spain; 3 ISPA (Instituto de Investigación Sanitaria del Principado de Asturias), 33011 Oviedo, Spain

**Keywords:** lipid oxidation, protein oxidation, natural antioxidant

## Abstract

Synthetic antioxidant food additives, such as BHA, BHT and TBHQ, are going through a difficult time, since these products generate a negative perception in consumers. This has generated an increased pressure on food manufacturers to search for safer natural alternatives like phytochemicals (such as polyphenols, including flavonoids, and essential oils rich in terpenoids, including carotenoids). These plant bioactive compounds have antioxidant activities widely proven in in vitro tests and in diverse food matrices (meat, fish, oil and vegetables). As tons of food are wasted every year due to aesthetic reasons (lipid oxidation) and premature damage caused by inappropriate packaging, there is an urgent need for natural antioxidants capable of replacing the synthetic ones to meet consumer demands. This review summarizes industrially interesting antioxidant bioactivities associated with terpenoids and polyphenols with respect to the prevention of lipid oxidation in high fat containing foods, such as meat (rich in saturated fat), fish (rich in polyunsaturated fat), oil and vegetable products, while avoiding the generation of rancid flavors and negative visual deterioration (such as color changes due to oxidized lipids). Terpenoids (like monoterpenes and carotenoids) and polyphenols (like quercetin and other flavonoids) are important phytochemicals with a broad range of antioxidant effects. These phytochemicals are widely distributed in fruits and vegetables, including agricultural waste, and are remarkably useful in food preservation, as they show bioactivity as plant antioxidants, able to scavenge reactive oxygen and nitrogen species, such as superoxide, hydroxyl or peroxyl radicals in meat and other products, contributing to the prevention of lipid oxidation processes in food matrices.

## 1. Introduction

The various food types present in the human diet primarily include meat, fish, fruits and vegetables and plant oils used for cooking. All of these provide essential nutrients due to their high amounts of polyunsaturated lipids, valuable proteins, essential amino acids, B-group vitamins and minerals, among others [1,2]. However, some of these food products (such as red meat or certain fish types) are prone to chemical and microbiological spoilage even under normal conditions of manipulation, processing and storage [3]. In fact, about 90 million tons of food are wasted every year in the EU, with almost half being discarded at retail and consumer levels for aesthetic reasons (e.g., discoloration) [4,5]. Lipids, especially polyunsaturated fatty acids (PUFAs), are the principal target of oxidative reactions, a key issue for both natural and processed foods. Proteins and carbohydrates can also be affected by oxidative reactions but to a lesser extent [6,7]. Food oxidation derived from altered lipids and proteins causes off-odors and off-flavors and color and textural changes, which not only decrease the sensory and nutritional quality of food products, but also their safety for the consumer due to the production of potentially toxic chemical compounds, such as polyoxygenated compounds derived from cholesterol in animal food matrices [8,9]. All these reasons make food oxidation a considerable challenge for the food preservation industry, since it decreases general product acceptance by consumers [10]. In this work, the different processes associated with oxidation in food matrices are reviewed and plant alternatives (flavonoids and terpenoids) to current synthetic antioxidant use as food additives are described for diverse food sectors.

## 2. Legislative Framework for Antioxidants in the Food Industry

The Codex Alimentarius Commission is the international standard-setting body responsible for the regulation and establishment of the set of food standards regulating the use of food antioxidants. These standards are neither mandatory nor directly applicable, as food regulatory systems and legislative frameworks in relation to the use of antioxidants as food additives vary between countries [11]. The two main guidance bodies overseeing the approval of food additives in the world are the European Food Safety Authority (EFSA) in the European Union and the Food and Drug Administration (FDA) in the United States of America [12,13]. 

Due to the growing interest in natural food antioxidants, many scientists argue that there should be a movement away from synthetic antioxidants, but this has not yet happened, and therefore the natural or synthetic origin of the different antioxidants currently used in food industry is not specified in the official tables showing the amounts and permissions for use of each additive in each type of food. In the EU, regulation EC No 1129/2011 establishes the list of antioxidants, classified according to their E-numbers, in the category of ‘other food additives’ in which their use is regulated. According to this regulation, extracts of rosemary (E392), ascorbic acid (E300) and tocopherols (E306–E309) are the natural antioxidants authorized as food additives by the EU [14]. In the case of the United States of America, food products and ingredients are regulated by multiple agencies, the main one being the U.S. Food and Drug Administration (FDA) [15]. The regulations concerning food products that fall under the purview of the FDA are found in Title 21 of the CFR of the U.S. Code of Federal Regulations (21 CFR 182; 21 CFR 172) and as in the case of European regulations, there is no specific category for natural antioxidants [16,17]. Although antioxidants approved for their use in food are clearly specified for such use and include natural antioxidants such as ascorbic acid or tocopherols, the US regulation is much broader than the European one and presents other ingredients that fall into other categories under a different technical effect, but which are known to possess proven antioxidant activity [18]. Examples include many of the ingredients approved for use as ‘coloring adjuncts’, ‘spices’ or ‘natural flavorings’ that have known antioxidant potential, such as flavonoids like phloretin glycoside, carotenoids like β-carotene and astaxanthin or extracts of rosemary or sage. In the specific case of phloretin, astaxanthin and sage extract, they are not included in European regulations for use as food additives in any category [19], but they are included in US regulations for other technical uses [18]. 

The perfect natural antioxidant for each food matrix is something that needs to be evaluated on a case-by-case basis, as in vitro antioxidant activities are not always reproduced in the food itself, as frequently there are alterations due to food processing or interactions with other food matrix components with antioxidant or prooxidant activities [20]. 

## 3. Oxidation Processes in Food Matrices 

### 3.1. Synthetic Antioxidants in Food Industry

If lipid oxidation is monitored over time in a given food matrix, a lag phase is usually observed, in which the accumulation of lipid oxidation byproducts is low. This lag phase is a result of, on the one hand, the low free radical formation that precedes the accumulation of hydroperoxides and ß-scission reactions and, on the other hand, the presence of antioxidants in the food matrix, which prevent the formation of free radicals that attack fatty acids. The aim of the food industry is to maximize the duration of this lag phase in which the concentration of the products responsible for rancidity taste are below human detection levels [20]. The most common solution used by food manufacturers to control this oxidation is the addition of antioxidants directly to the food matrix, when it is not rich in natural antioxidants and/or contains high endogenous prooxidants [20]. The term ‘antioxidant’ is commonly used in food science to denote compounds that block lipid peroxidation and other oxidative reactions, maintaining the freshness and prolonging the shelf-life of food products [21]. Whether the antioxidant is natural or synthetic, the mechanism of action is the same and includes free radical-scavenging, metal chelating and singlet oxygen quenching, among others [20]. 

**Figure 1:**
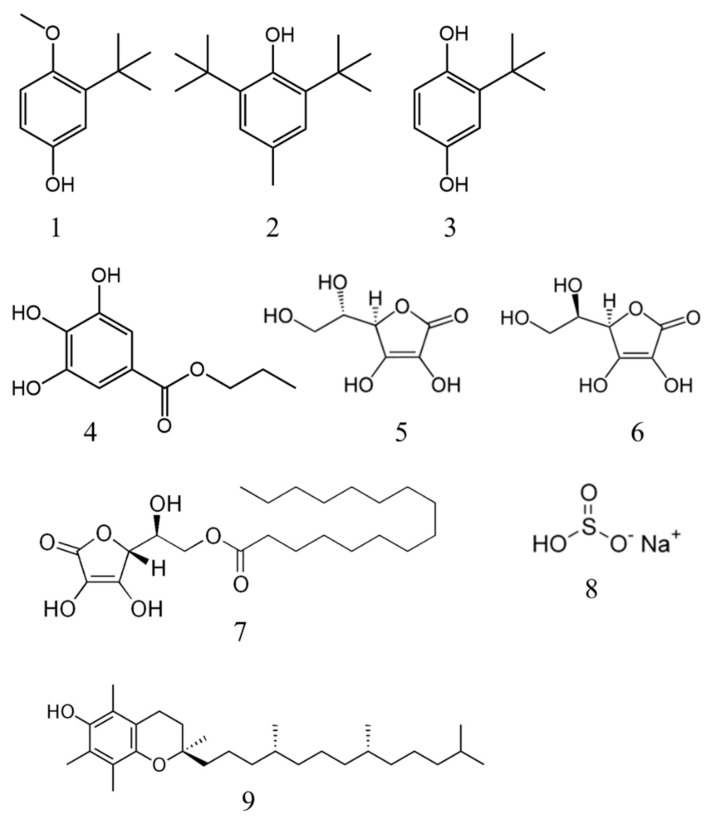
Chemical structures of food industry antioxidants. **1**: BHA, **2**: BHT, **3**: TBHQ, **4**: PG, **5**: ascorbic acid (vitamin C), **6**: erythorbic acid, **7**: ascorbyl palmitate, **8**: sodium bisulfite, **9**: vitamin E (alpha-tocopherol).

Synthetic antioxidants are widely used on the basis of their stability, low cost and high availability. The most widely used synthetic antioxidants in the food industry are phenolic antioxidants, among which the most important are: butylated hydroxyanisole (BHA) (E320), butylated hydroxytoluene (BHT) (E321), propyl gallate (PG) (E310) and tert-butyl hydroquinone (TBHQ) (E319) (Figure 1). In fruits and vegetables, 2-naphthol (2NL), 4-phenylphenol (OPP) and 2,4-dichlorophenoxyacetic acid (2,4-DA) are the most commonly used [22]. Although these synthetic antioxidants are widespread and strictly regulated, there are safety concerns due to their overuse and/or misuse (e.g., a mixture of certain antioxidants may increase their toxic effects) [23,24]. An increasing number of studies conclude that high doses of chemical synthetic antioxidants may cause DNA damage [25,26,27] or in vitro toxicity in certain tissues [28,29]. BHA functions as an environmental hormone and has a role as an endocrine disruptor. The effect of different concentrations of BHA (10–100 µM) in mouse testis cell lines (Leydig and Sertoli cells) was studied for its potential impact on human health, as there are many similarities between rodent and human steroidogenesis. It was found that this synthetic antioxidant induced cell cycle arrest in Leydig and Sertoli cells, inducing calcium dysregulation, endoplasmic reticulum stress and downregulating effects in steroidogenesis and spermatogenesis-related genes. This suggests a possible negative effect on male fertility [28]. The mechanism by which BHA induces its toxic effect on these cells is not yet clear, although a recent study suggests that its toxic activity may be attributed to increased levels of intracellular Ca^2+^ and Zn^2+^, resulting in increased sensitivity of rat thymocytes to oxidative stress [30]. In another study, BHT at 100 µM was added to mouse Leydig cells, causing suppression of cell proliferation, cell cycle arrest induction and effects on mitochondrial and endoplasmic reticulum calcium homeostasis [29]. Furthermore, BHA and BHT have been shown to be directly involved in the process of carcinogenesis in animal models [31,32]. 

Based on all of this evidence, the food industry is trying to decrease the use of these synthetic compounds by replacing them with natural alternatives in order to respond to the concern of consumers that they are being exposed through their daily diet to possibly dangerous chemical synthetic compounds. However, it is important to note that, although plant antioxidants are of natural origin, this fact does not make them safe by default; toxicity studies are always necessary for each particular case [22]. 

### 3.2. General Considerations Regarding the Antioxidants Mode of Action

In lipid-containing foods, antioxidants both delay the onset of oxidation and reduce the rate at which oxidation occurs. An ideal antioxidant should be inexpensive, non-toxic, effective at low concentrations, stable, able to endure food processing and at no time alter the sensory characteristics of the food, such as color, taste or odor [33].

Antioxidants can be classified as primary or secondary. Primary antioxidants are free radical scavengers that inhibit or delay initiation and/or propagation by donating electrons or hydrogen. Primary antioxidants (AH) react with lipid and peroxyl radicals (ROO^•^, RO^•^ and R^•^) and convert them into more stable products (ROOH, ROH and RH) by donating hydrogen atoms and generating antioxidant radicals in the process (A^•^), which are more stable and less reactive since the antioxidant radical is stabilized by delocalization of the unpaired electron along the phenolic ring to generate stable resonance hybrids. In addition, a dimerization process can also occur whereby the phenolic antioxidant radicals readily undergo termination reactions. This group of primary antioxidants includes phenolic compounds, tocopherols and synthetic antioxidants such as BHA, BHT, TBHQ and PG (Figure 1). 

On the other hand, secondary antioxidants do not convert free radicals into more stable products, but act indirectly by decreasing the rate of oxidation by chelating prooxidant metals, replenishing hydrogen to primary antioxidants, absorbing ultraviolet radiation or by functioning as singlet oxygen quenchers. Examples of secondary antioxidants are ascorbic acid (vitamin C), ascorbyl palmitate, erythorbic acid (a stereoisomer of vitamin C) and sulfite (Figure 1). 

**Figure 2:**
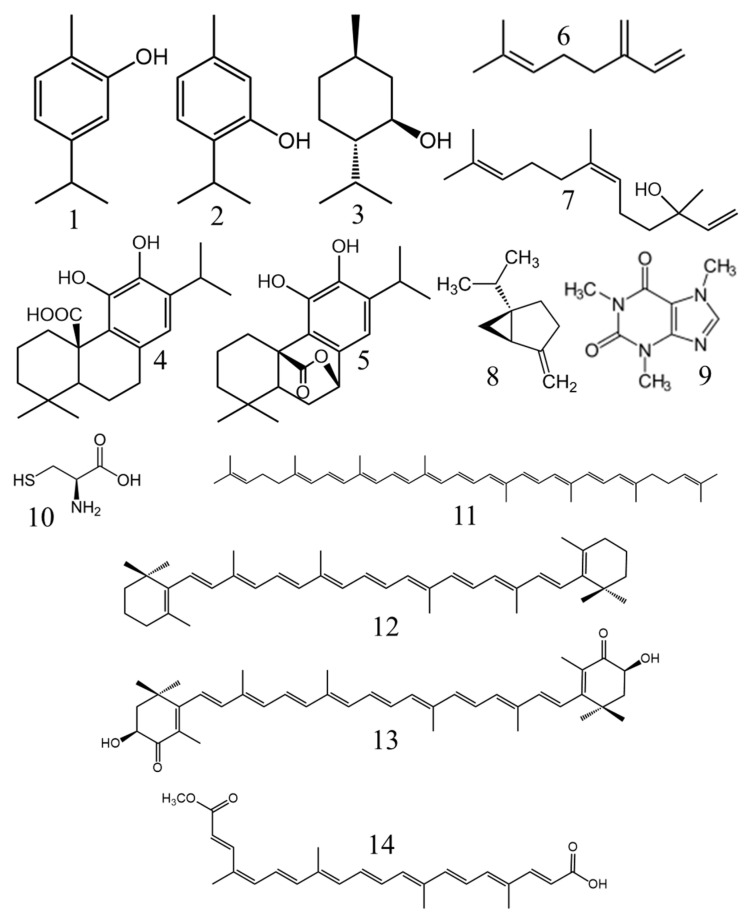
Chemical structures of plant terpenoid (including carotenoids) and nitrogen-containing antioxidants. **1**: carvacrol, **2**: thymol, **3**: menthol, **4**: carnosic acid, **5**: carnosol, **6**: myrcene, **7**: nerolidol, **8**: sabinene hydrate, **9**: caffeine, **10**: L-cysteine, **11**: lycopene, **12**: β-carotene, **13**: astaxanthin, **14**: bixin.

Carotenoids such as ß-carotene, lycopene or lutein (Figure 2) act as singlet oxygen quenchers, which deplete singlet oxygen of its excess energy and dissipate it as heat, thus returning it to an unexcited state and allowing the carotenoid to be recycled as an antioxidant. Citric acid and EDTA act as metal chelators (copper and iron), as these metals are involved in lipid oxidation. These two chelators also have a synergistic effect with phenolic antioxidants [11,33,34,35].

### 3.3. Lipid Oxidation Mechanisms

**Figure 3:**
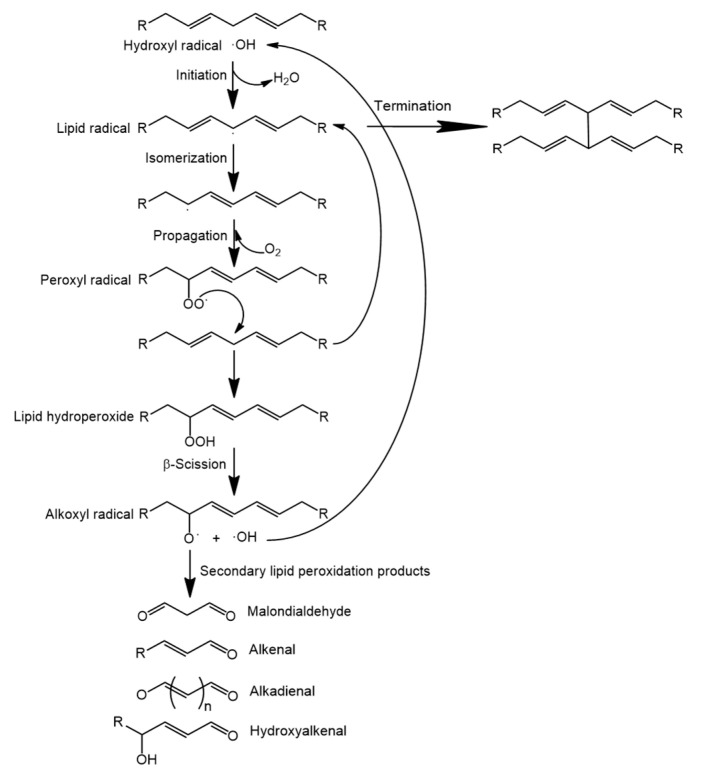
Oxidative processes involving polyunsaturated fatty acids and secondary lipid peroxidation products generated, involved in rancid odor.

Lipid oxidation occurs routinely in food matrices via free radical-mediated mechanisms, triggered by environmental and chemical factors, such as temperature, composition in unsaturated fatty acids, oxygen concentration and natural antioxidants present in the food matrix [36]. This process comprises three different phases: initiation, propagation and termination [21]. Initiation commences when a hydrogen is abstracted from an unsaturated fatty acid (RH) by a reactive species (^•^OH is the most reactive ROS and it is regarded as a potent initiator of lipid oxidation, ultimately being converted into H_2_O), thereby generating an alkyl radical (R^•^). In general, abstraction occurs in a methylene-interrupted carbon (a -CH_2_- moiety placed in the middle of two -CH=CH- moieties) of a PUFA (abundant in some meat and fish products and in some vegetable oils), in which the covalent bond strength between the hydrogen and its methylene carbon is reduced (Figure 3). The more double bonds contained in the fatty acid, the higher the susceptibility to oxidation due to the addition of more methylene-interrupted carbon reaction sites; in fact, the addition of a double bond to a PUFA doubles the rate of oxidation of the fatty acid. Enzymes, metals, metalloproteins, light and high processing temperatures act as catalysts of lipid oxidation in the initiation phase by generating reactive oxygen species that accelerate the oxidation process, such as the superoxide anion radical (O_2_^•−^), singlet oxygen (O_2_), the hydroxyl radical (^•^OH), the lipid radical (RO^•^) or the peroxyl-lipid radical (ROO^•^) [20,33,37]. Once the first hydrogen from the fatty acid has been abstracted, the energy of the generated alkyl radical is reduced by isomerization to form conjugated double bonds (one of the two former double bonds flanking the initially altered methylene-interrupted carbon is rearranged, generating two conjugated double bonds).

Initiation is followed by propagation, in which the alkyl radical reacts with atmospheric oxygen (O_2_) to form a peroxyl radical (ROO^•^) that has sufficient energy to promote the abstraction of a new hydrogen from another unsaturated fatty acid, thereby forming another alkyl radical (R^•^) and a lipid hydroperoxide (ROOH) (Figure 2). Hence, the propagation phase involves the transfer of a free radical from one fatty acid to another. The primary lipid peroxidation product that is generated is not volatile and does not contribute to the generation of rancid aromas; however, ß-scission reactions promoted by heat, light or transition metals, result in the decomposition of this lipid hydroperoxide (ROOH) through its reduction by an agent, such as ferrous iron (Fe^2+^), to produce two new radicals: an alkoxyl radical (RO^•^) and a hydroxyl radical (^•^OH). This generates a new free radical that can attack new fatty acids and leads to an exponential increase in lipid oxidation rates. In addition, alkoxyl radicals have high energy and are capable of breaking the aliphatic chain of the fatty acid, generating secondary lipid peroxidation products such as volatile hydrocarbons, short chain fatty acids, free radicals and low molecular weight molecules (aldehydes, ketones, hexane and malonaldehyde). In particular, these low molecular weight molecules are associated with the characteristic rancid odor of oxidized fats that can be detected by humans at the parts per million thresholds. Aldehydes (e.g., alkenals, alkadienals and hydroxyalkenals) are key secondary lipid peroxidation products due to their fast reactions with proteins, resulting in undesirable modifications in food [11,13,20,33]. 

In the termination phase, two radicals react to generate a radical-free molecule such as dimers, trimers or fatty acid polymers that precipitate, generating an increase in viscosity. The termination phase is not as important since the food already contains the secondary oxidation products (stable and non-reactive) that favor the formation of the rancid odor [13,20]. 

As far as lipid oxidation reactions are concerned, it is important to mention cholesterol (C_27_H_46_O), which is an essential component of the lipid fraction of food matrices from animal origins (such as red meat), as it is the most abundant lipid in eukaryotic cells. In food matrices, its oxidation occurs in a similar way to the oxidation of unsaturated fatty acids, but it should not be underestimated, as it results in the formation of mono- or polyoxygenated compounds (POPs), with deleterious effects on human health, such as atherogenic agents or proinflammatory triggers [38,39]. Additionally, the presence of unsaturated fatty acids in the food matrix accelerates the oxidation of cholesterol by creating a prooxidant environment, although it has been shown that lipids may compete for oxygen with sterols by reducing the rate of oxidation of the latter [35].

### 3.4. Protein Oxidation Mechanisms

Proteins are also susceptible to oxidation reactions both in the amino acid side chains and in the protein chain, which also alters the functional properties and thus the food quality. These changes are the result of physical and chemical reactions between proteins and are influenced by the presence of free radicals (e.g., ROS), non-radical species (e.g., H_2_O_2_ and ROOH) and myoglobin or metal catalysts. In addition, heat treatment also triggers oxidation, being responsible for protein denaturation as well [11,13,37]. Protein oxidation promotes the formation of carbonyls, hydroperoxides and sulfoxides, but it also causes fragmentation by scission of peptide linkages and protein aggregation due to cross-linking via L-cysteine or L-tyrosine residues; all leading to a reduction in protein solubility. These oxidation processes have, for example, a great impact on meat products by directly affecting their juiciness, tenderness, color changes, loss of protein digestibility or loss of protein functionality [13].

Protein carbonylation is the most important consequence of protein oxidation and occurs by a mechanism that shares many similarities with lipid oxidation. Firstly, during initiation, a hydrogen is abstracted from a protein (PH) by ROS (^•^OH radical mainly) to form a protein free radical (P^•^) and water. In the presence of O_2_, this protein radical is converted into a peroxyl radical (POO^•^), which reacts with another protein molecule (PH) and then a hydrogen is abstracted to form an alkyl peroxide (POOH) and a protein radical (P^•^). POOH can react with free hydroperoxyl radical (HO_2_^•^) or with reduced forms of transition metal ions (Mn^+^, Fe^2+^) to form an alkoxy radical (PO^•^). In the absence of O_2_, two protein carbon-centered radicals (P^•^) can react with each other to produce carbon–carbon cross-linked derivatives [13,37,40]. 

## 4. Methods Used for Antioxidant Evaluation in Food Model Systems

The evaluation of the oxidative stability of unsaturated fatty acids in food requires the measurement of their degradation over time (Tables 1–3). The methods designed for the evaluation of this stability can be grouped into two approaches. On the one hand, rapid tests try to expose the oil or fats samples to extreme conditions such as high temperatures for short periods of time in order to monitor their degradation. On the other hand, slow tests try to keep the sample in a highly controlled environment, and the oxidation processes are followed over time with different physicochemical methods that will be discussed below [41,42]. 

As discussed in Section 3.3, the compounds resulting from lipid peroxidation are known as primary or secondary lipid oxidation products. While primary ones (such as hydroperoxides) are not sensorially active and indicate early degradation, secondary lipid peroxidation products drastically decrease the sensory perception of the food. Two of the most commonly used methods to measure primary oxidation products are the peroxide value (PV) and conjugated dienes and trienes, while the most common methods to measure secondary oxidation products are the *p*-anisidine test or the 2-thiobarbituric acid reactive substance test (TBARS) [41].

The peroxide value test is one of the most common and simple tests to determine the primary oxidative status of high-unsaturated fatty acids oils [43] and relies on the separation of I_2_ from KI in the presence of hydroperoxides, which produces a yellowish hue that is indicative of the presence of hydroperoxides in the sample. Its low cost and ease of use make this method the most popular one in the food industry and it is routinely included in quality control specification sheets for certain oils. The PV is calculated and reported as oxygen milliequivalents per kilogram of sample (meq/kg). However, the PV value should be interpreted with caution, as a low value could be indicative of the absence of hydroperoxides or it could also indicate that these hydroperoxides have already been formed previously and have favored the formation of secondary oxidation products. For this reason, tests that measure these secondary products are also very interesting [41,42,44]. 

The TBARS test has been a very prominent assay since the 1960s. It uses 2-thiobarbituric acid (TBA) as a reagent that interacts with malondialdehyde and malondialdehyde-type products through a colorimetric reaction that generates a pink complex, which can be easily measured in a spectrophotometer with an absorption maximum of 530–535 nm [44]. This method is very popular in muscle foods (fish and meat) but has limitations in certain types of oils rich in oleic and linolenic acid, where malondialdehyde is a minority compound after the oxidation process. For this reason, it is a ubiquitous method in meat, but in the case of oils, it is preferable to measure primary oxidation (PV). TBARS are reported as malonaldehyde or malondialdehyde equivalents [41,42]. 

Another strategy to monitor oxidative processes involves the direct measure of free radical species, in order to quantify their inhibition due to the presence of antioxidants in the sample. This is the case of the DPPH radical (diphenylpicrylhydrazyl radical), which is stable at room temperature and absorbs at 517 nm, but can be reduced in the presence of an antioxidant molecule, generating a loss of color in the solution, which is then quantified. The results are shown as EC50, i.e., the amount of antioxidant required to decrease the initial DPPH concentration by 50% [44].

As far as protein oxidation is concerned, it also has an important impact on the sensory properties of the food due to the generation of small-molecular aldehydes formed during oxidative cleavage of amino acid side chains of proteins by ß-scission processes; however, the implications of these reactions on the taste of the food are still not entirely clear. In fact, there are far fewer examples of off-flavor formation due to protein oxidation compared to lipid peroxidation [45]. The most extensively used method to quantify protein carbonylation uses DNPH (2,4-dinitrophenylhydrazine) as a reagent to modify the carbonyl groups present on proteins towards the corresponding hydrazone adducts, which are detected spectrophotometrically at 375 nm. Here, the total protein concentration is determined at 280 nm using bovine serum albumin (BSA) as a standard. The results are usually expressed as nanomoles of carbonyls per milligram of protein [45,46].

## 5. Plant Antioxidants 

The plant kingdom is the major source of natural antioxidants, including pigments or products of plants secondary metabolism, which are generally derived from defense reactions against environmental aggressors. These bioactive compounds present in plant extracts have similar antioxidant capacity when compared to synthetic ones [35], with the added value of playing an important role in the chemoprevention of certain types of diseases. In fact, polyphenols, as an example of plant antioxidants, have a wide range of pharmacological and therapeutic properties, including anticancer, anti-inflammatory, antioxidant and vascular protective properties [47,48]. As far as natural antioxidants of plant origin are concerned, some of the most important groups are polyphenols, carotenoids and vitamins. These different classes of antioxidants are discussed below.

Polyphenols are derived from the secondary metabolism of most plants and can be divided into different classes depending on their chemical structure: phenolic acids (such as hydroxybenzoic acids and hydroxycinnamic acids), coumarins, lignans, chalcones, flavonoids, lignins and stilbenes [49]. Polyphenols of natural origin can be used to control oxidation processes in different food matrices and the most commonly used are leaf extracts from rosemary (*Salvia rosmarinus* L.) and sage (*Salvia officinalis* L.) and leaf and inflorescence extract from oregano (*Origanum vulgare* L.). Nevertheless, rosemary extract is the only one included in the European list of food additives with the number E392 (EU Regulation EC No 1129/2011), even though it has been shown that sage extract has a higher antioxidant potential [12].

The antioxidant potential of phenolic compounds depends on the number and position of the hydroxyl groups present in their molecules, which defines their scavenging potential of reactive radicals. For this reason, polymeric structures with a high number of hydroxyl groups show a higher antioxidant potential [35]. The molecular basis underlying the antioxidant activity of polyphenols can be attributed to two main mechanisms: direct reaction with free radicals acting as primary antioxidants or chelating free metals and therefore working as secondary antioxidants. The primary antioxidant effects of polyphenols are associated with their ability to inactivate free radicals by mechanisms involving the transfer of a hydrogen atom (HAT) or a single-electron (SET) to the free radical (R^•^) thereby stabilizing it. In the HAT mechanism, the phenolic antioxidant (ArOH) reacts with the free radical (R^•^) by transferring a hydrogen atom to it by breaking the O-H bond. The products of this reaction are the inactive RH species and the oxidized ArO^•^ radical, which is much more stable than R^•^. The free radical can also be stabilized by the SET mechanism via electron donation to the R radical, which results in an energetically stable R^−^ anion and a radical cation ArOH^•+^, which is then deprotonated by interacting with water. Both ArO^•^ and ArOH^•+^ radicals are aromatic structures in which the free radical has the possibility to move throughout the molecule resulting in its stabilization. In the particular case of flavonoids, the presence of a catechol group in the B-ring is the most decisive feature for scavenging reactive species derived from nitric oxide (RNOS), due to the catechol ability to donate hydrogen [33,34,50]. 

Carotenoids are tetraterpenes commonly used in the food industry and can be divided into two main groups: hydrocarbon carotenoids or carotenes (e.g., lycopene and ß-carotene) and oxygenated derivatives or xanthophylls (e.g., lutein) [35]. Lycopene (E160d) is the most widely used carotenoid as a food additive (food color) in the food industry and it is added to a wide variety of foods such as dairy products, fruits, meat, fish and sauces. Lutein (E161b) is another carotenoid with antioxidant properties that is widely used in the cosmetic industry for its high inhibition of lipid oxidation in skin cells. However, although EFSA allows its use as a food coloring agent, its use as an antioxidant is not allowed. On the other hand, astaxanthin has been extensively studied for its incorporation as a food preservative on the basis of its potent antioxidant activity, which is even higher than the antioxidant activity attributed to ß-carotene or α-tocopherol (vitamin E). The main disadvantage limiting the use of carotenoids as antioxidants is their high rate of oxidation in contact with light, posing a genuine problem with maintaining their stability during food matrix storage [12,35]. Consequently, the use of carotenoids as antioxidant agents in food is limited and they are not included as antioxidants in EC regulation No 1129/2011, although they are accepted as coloring agents [33,50]. 

Carotenoids are important agents as dyes and secondary antioxidants due to the large number of conjugated double bounds of the polyene chain that make radical scavenging possible, scavenging singlet oxygen and peroxyl radicals by physical quenching. Since singlet oxygen is one of the most reactive oxygen species (ROS) in cholesterol oxidation, carotenoids are particularly useful in preventing an oxidative attack of this sterol by singlet oxygen [35].

Apart from these two major groups (polyphenols and carotenoids), some minerals (Se and Zn) and vitamins (vitamin E and vitamin C) serve as cofactors of antioxidant enzymes and are therefore considered as natural antioxidants. Vitamin E encompasses tocopherols (E306–E309), which are phenolic compounds synthesized by plants, consisting of a chroman head conjugated with a phytyl chain. Their antioxidant activity consists of the donation of a hydrogen to a peroxyl radical, which is converted into unreactive hydroperoxide. In addition, tocopherols function as singlet oxygen scavengers. The oxidation products of vitamin E are considered to be prooxidants, actively involved in the propagation phase, requiring reduction (regeneration) by ascorbic acid, although it also regenerates carotenoids with greater affinity. Vitamin C or ascorbates (E300–E304) are considered to be the most potent hydrophilic antioxidants, acting in sequestration of radical superoxide anions, hydroxyl radicals, hydrogen peroxides, RNOS and singlet oxygen. It also acts as a reducing agent due to the four hydroxyl groups it has in its structure, which are responsible for donating hydrogen [11,12,35]. As a result of the excellent ability of ascorbic acid to regenerate other antioxidants, it is widely used in combination with antioxidants of synthetic origin such as BHT and BHA. Both tocopherols and ascorbic acid can be used as food antioxidants based on EU regulations [12].

### 5.1. Natural Antioxidants for Meat Food Matrices

Meat and meat products are an essential part of human nutrition as a source of proteins, amino acids, fats, minerals, vitamins and other nutrients. In 2015, the International Agency for Research on Cancer (IARC) declared that red meat was a probable human carcinogen (Group 2A), while there was sufficient evidence to declare that consumption of processed meat was carcinogenic to humans (Group 1) [51]. Since then, dietary trends have emerged in Western societies promoting the reduction or replacement of red meat in the diet. Despite this evidence, in most countries meat consumption has increased significantly over the last 20 years, reaching 360 million tons annually, with only 54% of this increase due to population growth [11,52]. Europe has not been an exception and it has been recently seen that, while in the 1960s the main protein source was derived from plant-based products such as wheat, currently 58% comes from animal-based products (28 g of protein/person/day), accounting for 30% of the calories ingested [53]. An interesting study focusing on the 2014–2016 timeframe concluded that the total per capita meat consumption was 34.1 kg/year, with about 60% being red meat (pork, lamb and beef) [54]. If analyzed from an economic point of view, the global meat sector was valued at 945.7 billion U.S. dollars in 2018 and it was forecasted to increase to 1142.9 billion U.S. dollars by 2023 [55]. Therefore, the high daily consumption rates associated with meat products make it a group of special consideration for replacing synthetic antioxidants with those of natural origin.

Lipid and protein oxidation of meat products is the second most important cause of meat spoilage (after microbial spoilage), generating a negative impact on the organoleptic features of the product [11]. Moreover, oxidative processes of cholesterol generate POPs derivatives, which are associated with deleterious effects on human health, such as inflammation [56,57,58], cytotoxicity [59], carcinogenesis [60,61,62,63] or atherogenesis [57,64], and the development of degenerative diseases such as Alzheimer’s [58], Parkinson’s [65], Huntington’s [66] and other chronic diseases [58]. Lipid lipoperoxidation and oxidation of pigments (such as heme in myoglobin) and proteins (such as collagen) reduces the quality and nutritional value of meat products [40] and it is a process that begins during the conversion of muscle to meat products and continues during its processing and storage. Meat and meat products are highly susceptible to oxidation processes as they are rich in polyunsaturated fatty acids derived from phospholipids in membrane lipoproteins [13]. Additionally, the composition of meat has a substantial influence on its oxidative stability; in fact, red and darker meats have a higher concentration of myoglobin and consequently more heme pigments and reactive iron atoms, which are known catalysts of lipid oxidation [37]. Antioxidants used in the meat industry are both natural and synthetic and the natural antioxidants can be added directly or after extraction and purification of the antioxidant substances they contain [11]. However, regulation EC No 1129/2011 only allows the use of rosemary extracts for certain types of meat products [14].

Rosemary oil is an important source of natural antioxidants to be used in meat products, such as carnosic acid, carnosol or the monoterpene myrcene (Figure 2) (Table 1) [67]. It was reported that sage and rosemary essential oils exhibited similar protein antioxidant properties to BHT in refrigerated porcine liver pâté [46]. Essential oils have some sensitivity to environmental conditions (such as temperature, light and oxygen). In order to avoid these deleterious effects, and also evaporation, essential oils can be encapsulated [68,69]. Rosemary extracts have also been tested in lamb meat to stabilize the sensory quality of cooked and chilled lamb [67]. Oregano extract has a well-known antioxidant activity and it was demonstrated that its inclusion in the manufacturing of cooked sausage showed lower levels of lipid and protein oxidation for a storage time of 135 days, compared to the positive control with added sodium erythorbate [70]. One of its terpenoid components is carvacrol (Figure 2), which has shown significant antioxidant activity in burger meat, individually and combined with green tea extract, in contrast with sulfite-based synthetic preservatives [71].

**Figure 4:**
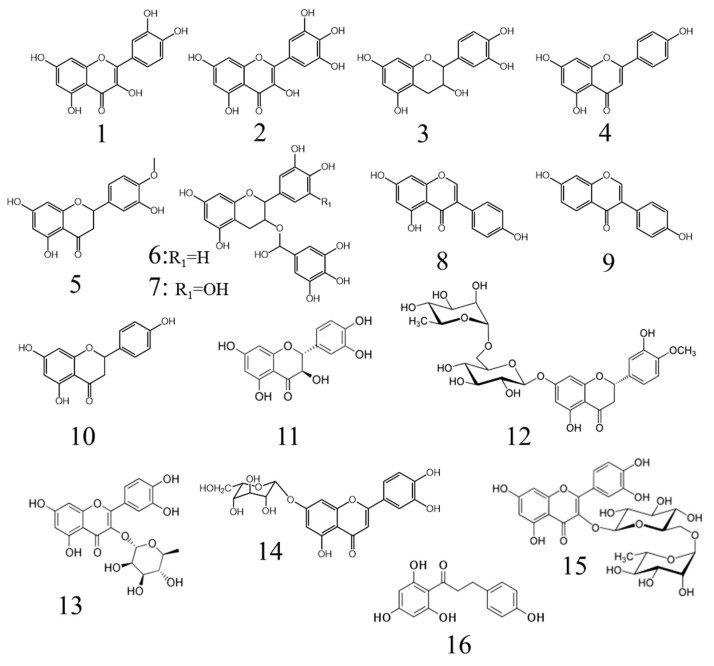
Chemical structures of flavonoid antioxidants. **1**: quercetin, **2**: myricetin, **3**: catechin, **4**: apigenin, **5**: hesperetin, **6**: epicatechin gallate, **7**: epigallocatechin gallate, **8**: daidzein, **9**: genistein, **10**: naringenin, **11**: dihydroquercetin (taxifolin), **12**: hesperidin, **13**: quercitrin, **14**: rutin, **15**: luteolin-7-*O*-glucoside, **16**: phloretin.

Rosemary and oregano extracts have classically been the target of numerous scientific studies, but other plant-derived extracts have also shown interesting features. Recently, different ethanolic extracts obtained from cork oak leaves (*Quercus suber* L.) with a rich composition in phenolic compounds (catechin, epicatechin, gallic acid, rutin, myricetin or quercetin) (Figure 4) were studied to control oxidation in a cooked chicken model (2% *v*/*w*) and showed high antioxidant activity after 5 days of storage at 4 °C. Compared to the control group, all tested conditions prevented lipid oxidation in an equivalent way to BHT added at 2% (*v*/*w*) [72]. *Chrysanthemum morifolium* Ramat. flower extract contains about seventy phenolic compounds, most of them being flavones, flavonols, isoflavonoids and flavonones, among others. Its effect on lipid and protein oxidation was studied when it was added at 0.1% and 0.2% to goat patties and stored under refrigeration for 9 days, using the same meat matrix but with 0.01% BHT as a control. The matrices with a higher percentage of flower extract had a higher antioxidant activity than the control group and protein oxidation was also reduced when the flower extract was added. In none of the cases were the sensory characteristics of the product affected [73]. In another study, guava leaf extract (*Psidium guajava* L.) was added at different concentrations to fresh pork sausages and compared to a control with added BHT. Both conditions were monitored for a total of 14 days at 4 °C and it was shown that the formation of primary and secondary products of lipid oxidation was retarded to the same extent as when the synthetic antioxidant was added to the meat [74]. A recent study focused on *Cymbopogon citratus* D.C. extract, which was analyzed in detail and showed numerous phenolic and flavonolic compounds. This gives an idea of its antioxidant activity, which was contrasted after being added to chicken sausage refrigerated for up to 42 days, producing a reduction in lipid oxidation with respect to the control treated with the synthetic antioxidant sodium erythorbate [75]. 

Guarana seed polyphenols can improve the preservation of redness during the storage of raw pork patties avoiding the formation of metmyoglobin (an oxidized version, which causes a brown color in red meat products) [76]. It was also found that grape seed extract can reduce the production of this oxidized form of myoglobin, slowing lipid oxidation during the storage of cooked chicken sausage at 4 °C for 40 days [77,78]. Regarding pomegranate, its lyophilized peel nanoparticles retarded lipid oxidation thus improving sensorial appearance, microbial quality and cooking characteristics of meatballs [79]. An extract of mesquite leaf resulted in a significant increase in antioxidant levels and color stability of pork patties and in lipid oxidation stability of treated pork patties during the storage time [80]. Black rice is another source of polyphenols, especially anthocyanins. A black rice extract slowed lipid oxidation in raw beef patties during 6 days of refrigerated storage [81].

**Figure 5:**
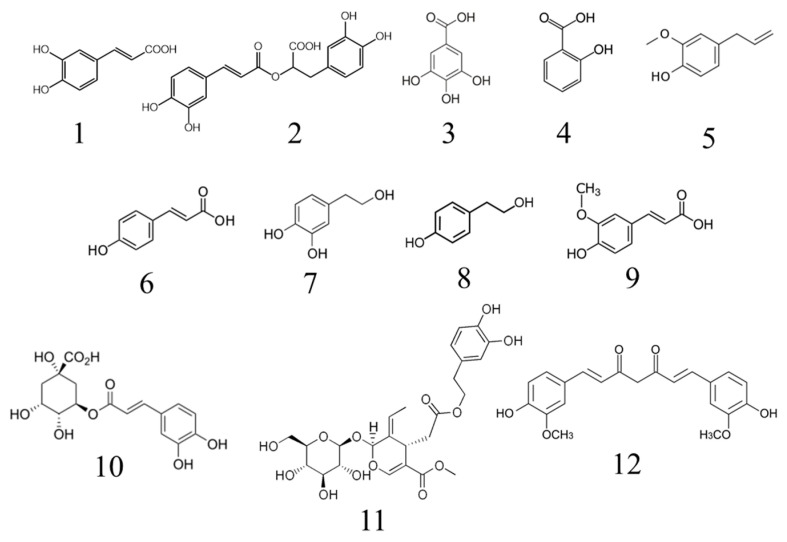
Chemical structures of phenolic acids antioxidants. **1**: caffeic acid, **2**: rosmarinic acid, **3**: gallic acid, **4**: salicylic acid, **5**: eugenol; **6**: p-coumaric acid, **7**: 4-hydroxytyrosol, **8**: tyrosol, **9**: ferulic acid; **10**: chlorogenic acid, **11**: oleuropein; **12**: curcumin.

Tea polyphenols contain catechins (85% of its polyphenol content), flavones, anthocyanidins and phenolic acids, which showed a powerful antioxidant activity and no toxicity [82]. In the case of green tea, its antioxidant activity is mainly due to the presence of catechins. On the other hand, catechin oligomers from apples and phenolic acids such as caffeic acid and gallic acid (Figure 5) are effective inhibitors of cholesterol oxidation in commercial meat products such as sausages, raw and roast ham, bacon, minced meat and hamburgers [83,84].

Honey is a rich source of polyphenols with known beneficial effects on human health, but there are not many studies on bee pollen. These pellets of field-gathered flower pollen packed by worker honeybees can be considered a functional food because they are rich in proteins, lipids, minerals and natural antioxidants, such as phenolic acids, flavonoids, sterols, carotenoids and vitamins. The antioxidant capacity of bee pollen (mainly composed of *Cistus ladanifer* L. pellets) to prevent lipid oxidation in black pudding (blood sausage) was recently studied. Three different mixtures were prepared, one with fresh pollen, one with an ethanolic extract of fresh pollen and one with sodium ascorbate as a control. The mixtures were then stored for 37 days and it was observed that bee pollen had a high-quality antioxidant activity comparable to sodium ascorbate [85].

Processed meat is widely known to be high in fat, saturated fatty acids, cholesterol, protein, salt and synthetic additives with deleterious effects on human health [51] and a transition to healthier processed meat products is necessary. The use of antioxidants in these food matrices is essential due to their high fat content, which makes them particularly sensitive to lipid oxidation. However, these types of products are subject to a strict judgement by consumers who are unwilling to accept reductions in the sensory quality of the product (color, smell or taste), which is often affected by the incorporation of natural antioxidants [86,87]. This problem could be partially solved by incorporating the antioxidant in the form of microcapsules [88]. In a recent study, the authors compared the sensory acceptance and antioxidant capacity of a cooked meat sausage to which a synthetic antioxidant was added and another to which curcumin (Figure 2) microcrystals were incorporated and stored for 90 days in the refrigerator at 4 °C. The inclusion of curcumin in the product significantly increased its antioxidant activity with respect to the synthetic control, but consumer acceptance of the product slightly decreased due to more yellowish coloration [88]. The reduction of the sensory quality of the processed food is highly dependent on the origin and amount of the natural antioxidant used and there are many examples in the literature where the incorporation of polyphenol-rich compounds such as pink pepper extract or kiwifruit extract to chicken burgers [89] and beef samples [90] respectively had a potent antioxidant effect after 7 days of refrigerated storage, without altering their sensory properties.

Microalgae are recognized sources of carotenoids, vitamins minerals that play an important role for their use as antioxidant compounds in meat matrices. However, the use of the complete microalgal biomass could negatively alter the organoleptic characteristics of the meat product, so the use of the isolated antioxidant compound could be a more suitable approach; unfortunately, this generates a significant increase in production costs [91]. Astaxanthin (Figure 2) is one of the most potent natural antioxidants and it is safe even in high doses, showing a higher antioxidant activity than α-tocopherol. For all these reasons, it is commonly used as a food supplement or in animal feed, but more and more studies are focusing on its use as a food additive to increase oxidative stability. A rich source of this carotenoid is the microalgae *Haematococcus pluvialis*, which was recently studied for the first time as a food preservative. For this purpose, its extract was added at different concentrations to raw ground pork meat and placed in storage for 7 days in a refrigerator. The addition of astaxanthin extract significantly delayed lipid oxidation without affecting the sensory characteristics of the product [92]. The antioxidant effect of this carotenoid was also tested in emulsified pork sausages during cold storage. The results showed that the matrix containing astaxanthin showed higher lipid and protein stability than the control and the effect was similar to that obtained with the positive control with added BHT [93]. 

It must be taken into account that large amounts of polyphenol antioxidants may be required to replace synthetic ones at an industrial level, extracted from large quantities of plant biomass. This economic problem could be solved by obtaining these polyphenols from agriculture waste materials, such as seeds and peels [94]. Within the framework of the circular economy, many efforts have been directed towards the use of natural additives from byproducts generated by the agrofood industry, a notable example being the tomato (*Solanum lycopersium* L.) industry. This industry generates large quantities of tomato pomace, which consists of a mixture of skins and seeds with a small fraction of pulp residues rich in carotenes (lycopene, ß-carotene or lutein) (Figure 2), phenolic compounds (flavonoids) and vitamins (ascorbic acid and tocopherols) (Figure 1). Among all of those, lycopene constitutes 80–90% of the total pigments and is of special interest to the meat industry, not only for its known in vitro antioxidant activity, but also its prevention of discoloration [95,96,97]. In this context, two concentrations of tomato powder (1% and 2%) were added to emulsified pork sausages and the antioxidant potential was similar to that obtained with 0.01% BHT during 28 days of storage at 10 °C [98]. Another example of byproduct, in this case from the olive processing industry, is olive pomace, which is a solid residue containing fragments of skin, pulp, pieces of kernels and some oil. Finally, grape pomace (seeds, stems and skins) is the main waste generated in the wine industry, particularly notable for its rich content in polyphenols, flavonoids and tannins. A study showed the effectiveness against lipid and protein oxidation of the latter two (olive and grape pomaces) when added (1000 mg/kg) into lamb meat patties [96]. Unfortunately, the number of studies on the use of these products in the meat industry is small compared to the number of publications on other plant extracts.

**Table 1:** Effect of natural antioxidants on lipid and protein oxidative stability of meat food matrices. MDA: malondialdehyde; N/A: not available; GAE: gallic acid equivalent; TPC: total phenolic content.

Product Used	Natural Source and Plant Organ	Extraction Method	TPC	Natural Bioactives Content	Substrate Concentration	Lipid/Protein Oxidation	Main Results	Reference
Porcine liver pâté	*Salvia rosmarinus* L. essential oil extract from commercial source	N/A	217 mg GAE/100 g	N/A	0.1%	<7 nM carbonyls/mg protein (DNPH) Percent inhibition of protein oxidation 51.28%	Similar antioxidant properties to BHT (0.02%) for 90 days storage under refrigeration at 4 °C	[46]
Porcine liver pâté	*Salvia officinalis* L. essential oil extract from commercial source	N/A	203 mg GAE/100 g	N/A	0.1%	<6 nM carbonyls/mg protein (DNPH) Percent inhibition of protein oxidation 59.66%	Similar antioxidant properties to BHT (0.02%) for 90 days storage under refrigeration at 4 °C	[46]
Sheep sausages	*Origanum vulgare* L. extract from whole plant	Solvent extraction (Acetone, water, glacial acetic acid)	517.21 mg GAE/g	N/A	6630.98 mg/kg–8038.20 mg/kg	1–2 mg MDA/kg sample	Oregano extract improved the lipid and protein stability of cooked sausages during 135 days at 20 °C in dark conditions compared to sodium erythorbate (500 mg/kg)	[70]
Lamb burger meat	Green tea extract and carvacrol from commercial sources	N/A	108.25 mg GAE/g green tea extract and 77.78 mg GAE/g carvacrol	N/A	300 ppm of green tea extract, 300 ppm of carvacrol	<0.50 mg MDA/kg meat	Both carvacrol and green tea extract avoided lipid oxidation and showed a lower content of MDA than sulfite treatment (400 ppm) for 8 days at 4 °C. Carvacrol brought herbal flavors to the meat	[71]
Cooked meat model (chicken)	*Quercus suber* L. leaf extracts	Solvent extraction (water and ethanol)	6.1–10.8 mg GAE/g	Phenolic acids, gallic acid, flavonols, flavanols, rutin, myricetin, quercetin, catechin, epicatechin	2% *v*/*w*	<0.50 mg MDA/kg meat	Cork oak leaf extracts prevented lipid oxidation and the ability to control oxidation was equivalent to BHT (2% *v*/*w*) in cooked chicken models during 5 and 10 days of storage at 4 °C	[72]
Goat meat patties	*Chrysanthemum morifolium* Ramat. flower extracts	Solvent extraction (methanol)	N/A	Gallocatechin, apigenin 7-*O*-glucoside, rosmarinic acid, rhamnetin, caffeic acid, 3-hydroxybenzoic acid, kaempferol 3-*O*-galactoside	0.1–0.2%	<1.4 mg MDA/kg meat and <3.5 nmol carbonyl/mg protein	Samples with 0.2% of extract showed the lowest ranges of TBARS and carbonyl content values followed by BHT (0.01%) during 9 days of refrigerated storage at 4 °C. Sensory qualities were not affected	[73]
Fresh pork sausage	*Psidium guajava* L. leaves extract	Solvent extraction (hydroalcoholic)	8.87 mg GAE/g	N/A	4000–6000 ppm on fat basis	<1 mg MDA/kg sausage	Sausage formulated with guava leaf extract were as effective as 200 ppm BHT treatment at slowing the process of lipid oxidation during 14 days of refrigerated storage at 4 °C	[74]
Chicken fresh sausage	*Cymbopogon citratus* D.C. leaf extract	Solvent extraction (hydroalcoholic)	133.84 mg GAE/g	Flavonoids (luteolin, amurensin, isoflavanone), phenolics (caffeic acid, mananthoside h, protocatechuic acid)	0.5%	0.21 mg MDA/kg	The extract reduced lipid oxidation compared to sodium erythorbate (0.1%) and the sensorial characteristics were maintained for up to 42 days of storage	[75]
Pork patties	*Paullinia cupana* Kunth seed extract	Solvent extraction (hydroalcoholic)	258 mg GAE/g	Tyrosols, phenolic acids, anthocyanins, alkylphenols, flavonols, flavones, stilbenes, lignans	500–250 mg/kg	0.08–0.07 mg MDA/kg and 2.47–3.13 nmol/mg	The antioxidant efficacy of the extract in protecting patties against lipid and protein oxidation was even more effective than the efficacy of the synthetic antioxidant BHT (200 mg/kg) when evaluated for 18 days of storage at 2 °C	[76]
Cooked chicken sausage	Cinnamon essential oil from bark of *Cinnamomum zeylanicum* J.Presl and commercial grape seed extract from *Vitis vinifera* L.	Hydrodistillation (cinnamon essential oil)	N/A	N/A	Cinnamon essential oil (0.04% *v*/*w*) plus grape seed extract (0.16% *v*/*w*)	2.91 mg MDA/kg	The combinatorial use of cinnamon essential oil and grape seed extract extended shelf-life of chicken sausage by retarding the lipid oxidation during 40 days of storage at 4 °C	[78]
Meatballs	*Punica granatum* L., cv. Manfaloty peel nanoparticles	N/A	215 mg GAE/g	Punicalagin, *p*-hydroxybenzoic, rutin, kaempferol, caffeic acid, ferulic acid, catechine	1–1.5%	1.03–0.77 mg MDA/kg	Pomegranate peel nanoparticles significantly reduced lipid oxidation when compared with BHT (0.1%) during storage at 4 °C up to 15 days, and they improved the sensorial acceptance the final product	[79]
Pork patties	*Prosopis velutina* Wooton leaves extract	Solvent extraction (hydroalcoholic)	278.5 mg GAE/g	N/A	0.05–0.1% *w*/*w*	<0.1 mg MDA/kg	The extract decreased the TBARS values 90% in comparison with the control group (without antioxidant) during 10 days of storage at 4 °C	[80]
Beef patties	*Oryza sativa* L. powder from commercial source	Aqueous extract	270.51 mg GAE/g	N/A	0.4–1.2%	<0.5 mg MDA/kg	The addition of black rice water extract decreased lipid oxidation and improved redness when compared with control (without antioxidant) during 6 days of storage under fluorescent light at 2 °C	[81]
Camel meat	Tannic acid and catechin from commercial source	N/A	N/A	N/A	200 mg/kg	<0.5 mg MDA/kg	Tannic acid and catechin treated samples could retard lipid oxidation and were also effective in maintaining sensorial quality of meat during 9 days of refrigerated storage	[84]
Black pudding	Fresh and bee pollen extract mainly composed by *Cistus ladanifer* L. pellets	Solvent extraction (hydroalcoholic)	35.05 mg GAE/g	Myricetin, luteolin and quercetin *O*-derivatives	N/A	1.30–1.50 mg MDA/kg	The black pudding prepared with pollen as the natural antioxidant showed similar TBARS values to those observed for the black pudding prepared with the commercial antioxidants (sodium ascorbate) and stored for 37 days at 4 °C	[85]
Mortadella	Microcrystals of curcumin from *Curcuma longa* L. (commercial source)	N/A	N/A	N/A	0.002%	1.11 mg MDA/kg	The mortadella with curcumin microcrystals showed significantly lower TBARS values at the end of the storage (90 days 4 °C) when compared to the standard treatment (addition of synthetic antioxidant). The addition of curcumin decreased the acceptance of color’s sample	[88]
Chicken burger	*Shinus terenbithifolius* Raddi extract	Solvent extraction (hydroalcoholic)	12.17 mg GAE/g	N/A	Equivalent to 90 mg GAE/kg meat	<0.3 mg MDA/kg	Pink pepper extract was as effective as BHT (90 mg/kg) in delaying lipid oxidation of a chicken burger after 7 days of refrigeration at 2 °C	[89]
Beef	Fruit extract from *Actinidia deliciosa* (A. Chev.) C.F. Liang and A.R. Ferguson	Solvent extraction (hydroalcoholic)	82.84 mg GAE/g	Epicatechin, catechin and quercitrin	100 mg/kg	0.25 mg MDA/kg	Samples treated with the fruit extract could significantly inhibit lipid oxidation without significant alteration of beef sensorial properties during storage in a refrigerator at 4 °C for 7 days	[90]
Ground pork meat	*Haematococcus pluvialis* extract from commercial source	N/A	N/A	5% of astaxanthin	0.3–0.45 g/kg	0.99–0.85 mg MDA/kg	The *Haematococcus pluvialis* extract delayed lipid oxidation and improved color stability with a positive effect on meat acceptance at the 7th day of refrigerated storage at 4 °C	[92]
Emulsified pork sausages	Astaxanthin from commercial source	N/A	N/A	Astaxanthin	400 mg/kg	0.16 mg MDA/kg	The emulsified sausages with added astaxanthin exhibited significantly higher redness values, greater acceptability, and similar lipid oxidation stability than BHT (200 mg/kg) on 21 days of storage	[93]
Raw lamb patties	Olive and grape pomace agroindustrial byproducts	Aqueous extract	85.41 mg GAE/g (olive pomace) and 32.16 mg GAE/g (grape pomace)	N/A	1000 mg/kg	1.37 mg MDA/kg (olive pomace) 1.28 mg MDA/kg (grape pomace)	Grape and olive pomace aqueous extracts delayed lipid oxidation throughout storage (7 days under refrigeration)	[96]
Pork sausages	Fruit powder from *Solanum lycopersium* L.	N/A	2.16 g GAE/100 g	N/A	1–2%	0.06–0.08 mg MDA/kg	Sausage samples with tomato powder showed lower TBARS values than the control (without antioxidants) and did not differ from reference sausage with BHT (0.01%) during 28 days of storage at 10 °C	[98]

### 5.2. Natural Antioxidants for Fish Food Matrices

Preservation of seafood is a real challenge, since it has a shorter shelf-life compared to chicken and red meat. Fish products are essential in a healthy human diet due to their high nutritional value, as they contain vitamins, trace-elements and macroelements and a high number of important enzymes, proteins and fatty acids. Lipid content is the main reason for their vulnerability to oxidation [99], as seafood is a rich source of PUFAs like omega-3 fatty acids [100]. Furthermore, fish products barely contain endogenous antioxidants in comparison to other food products [101]. Fish spoilage begins at the very moment of fish death due to microbial activity and autoxidation and there is a need for more suitable and efficient preservation methods using natural compounds to lengthen this short shelf-life and make its management easier [102].

Phytochemicals, mainly phenolic compounds, hold interesting potential applications in the seafood industry as bioactive compounds, contributing to the production of new functional foods. It has been shown that citrus peel is rich in phenolic acids and flavonoids and has a high antioxidant activity, even stronger than synthetic antioxidants. Fish meats enriched with extracts from cabbage leaves (*Brassica oleracea* var. *capitata* L.) and banana peels (*Musa* sp. L.) were studied for their oxidation stability. The phenolic antioxidants present in the extracts were able to interact with free radicals from lipid oxidation, blocking the deterioration process and improving lipid stability and also sensory acceptability for consumers [103]. 

The flavonoid phloretin (Figure 4) is the most abundant phenolic compound in apples (*Malus domestica*) and it is well known for its considerable antibacterial (mainly against Gram-positive bacteria) and antioxidant activity (Table 2). In fact, this compound is approved by the FDA and the maximum allowed level in foods is 100 ppm. Phloretin has been tested as a preservative in Atlantic salmon (*Salmo salar*) fillets and lipid oxidation was shown to be more than three times lower than in controls [104]. Atlantic mackerel (*Scomber scrombus*) is another highly exploited commercial fish and it has been proven that fillets, previously immersed in solutions of rosemary (*Salvia rosmarinus* L.) and basil (*Ocimum basilicum* L.) essential oils and stored at 2 °C, had their shelf-life prolonged for several days. The main rosemary compounds involved in antioxidant activity are carnosol and carnosic acid (Figure 2) [105], while in basil, eugenol (Figure 5) stands out [106]. These compounds, among others, inhibited the formation of lipid oxidation products, delaying degradation and maintaining fish meat quality both at molecular and perceptible levels [105]. It was also shown that the peel extract from the cactus pear (*Opuntia ficus-indica* Mill) has a high content of polyphenols, so it was selected to be used as a preservative in sardine (*Sardina pilchardus*) fillets during a long refrigerated storage period [107]. The fillets were soaked in an aqueous solution enriched with polyphenols and lipid oxidation was delayed due to the inhibition of bacterial metabolism and enzymatic degradation, without affecting sensorial aspects.

Processed fish products such as fish sausages have been the target of a recent study in which a tocopherol mix (Figure 1) was directly added to the meat via nanoemulsions [108]. This type of encapsulation not only made the dispersion easier, considering tocopherol’s lipophilic nature, but also increased its bioavailability. An increased oxidative stability and a better quality of the fish sausages during refrigerated storage was demonstrated, maintaining their organoleptic properties.

Phenolic compounds can also be obtained from waste materials. In this manner, filtered byproducts derived from *Rosa damascena* Mill. essential oil distillation were used for tissue impregnation of sea bass (*Dicentrarchus labrax*) fillets. This process produced an improvement in fish quality and oxidation stability, increasing its shelf-life when combined with osmotic treatment, which reduces the microbial spoilage [101]. Another example of circular economy comes from the brewing industry. Brewers’ spent grain (leftover malt) is usually discarded, but it is a product rich in flavonoids and phenolic acids, such as ferulic acid, *p*-coumaric acid and caffeic acid (Figure 5). Although these polyphenols cause an unpleasant bitter taste when added to fish products, a study focused on microencapsulating it into a modified starch and adding it to fish meat [109]. These microcapsules were tested in sea bass burgers and a more stable final product with a lower oxidation activity was obtained, even after cooking. The olive oil industry also produces a high number of byproducts; dry olive paste flour, rich in phenolic compounds (mainly tyrosol and hydroxytyrosol, caffeic acid and *p*-coumaric acid, Figure 5) was added to tuna (*Thunnus* sp.) burgers [110]. The dry paste was pretreated with milk to reduce the unpleasant taste and it increased the nutritional value of the fish burgers, since the polyphenolic content showed an 8× increase in comparison with the control, and with the antioxidant activity being enhanced as a result.

Another interesting way to increase fish meat quality is to include natural supplements in the fish diet. The sesquiterpene nerolidol (Figure 2) was included (free or nanoencapsulated) in the Nile tilapia (*Oreochromis niloticus*) diet [111]. In this experiment, both free and encapsulated nerolidol strengthened the antioxidant systems in the fish muscle. Growth rate and weight increased, unlike reactive oxygen species and lipid peroxidation, which were significantly reduced, improving overall fish meat quality.

**Table 2:** Effect of natural antioxidants on lipid oxidative stability of fish food matrices. MDA: malondialdehyde; N/A: not available; GAE: gallic acid equivalent; meq: milliequivalents; CATE: catechin equivalents; LPO: lipid peroxidation; ROS: reactive oxygen species; TPC: total phenolic content.

Product Used	Natural Source and Plant Organ	Extraction Method	TPC	Natural Bioactives Content	Substrate Concentration	Lipid Oxidation	Main Results	Reference
*Labeo rohita* meatballs	*Brassica oleracea* L. var. *capitata* leaves and *Musa* sp. L. fruit peels extracts	Aqueous extract	N/A	N/A	0.5, 1 and 1.5%	1.31–1.85 meq O_2_/kg (4 °C) 1.31–1.41 meq O_2_/kg (−18 °C)	The phenolic antioxidants present in the extracts improved lipids stability and sensory acceptability throughout refrigeration storage (at refrigerator for 9 days and at −18 °C in a freezer for a period of 60 days)	[103]
*Salmo salar* fillets	Commercial standard	N/A	N/A	Phloretin	2–4 mg/mL	1.7–1.3 mg MDA/kg	Lipid oxidation was reduced by 3.23 times compared to control samples (not treated) after 3 days of storage at 4 °C	[104]
*Scomber scombrus* fillets	*Salvia rosmarinus* L. and *Ocimum basilicum* L. essential oils	Hydrodistillation	N/A	Carnosol and carnosic acid Eugenol	1% *w*/*v*	5.60–4.20 mg MDA/kg	The basil and rosemary essential oils extended the shelf life of Atlantic mackerel fish by 2 and 3–5 days, respectively, compared to the control group (not treated) when stored at 2 °C for 15 days	[105]
*Sardina pilchardus* fillets	*Opuntia ficus-indica* Mill peel extract	Solvent extraction (hydroalcoholic)	1472 mg CATE/100 g	N/A	1/10 *w*/*v*	2.0 mg MDA/kg	The addition of a cactus extract extended the shelf life of sardine fillets without altering their sensorial properties after 11 days of storage at 2 °C	[107]
*Xenobrama microlepis* sausages	Commercial standard	N/A	N/A	Tocopherol mix	250–500 mg/kg	30.5–32.0 meq O_2_/kg	It was observed that fish sausages containing a tocopherol nanoemulsion were effective at delaying lipid oxidation compared to the control (not treated) during 16 days of refrigerated storage without affecting the final product texture or pH	[108]
*Dicentrarchus labrax* fillets	*Rosa damascena* Mill. essential oil from flowers	Hydrodistillation	1650 mg GAE/L	N/A	1500 ppm	1.3 μmol MDA/g	The shelf life of fish was extended significantly (four-fold increase) when impregnated with *Rosa damascena* phenolics as compared to the untreated samples during 30 days of storage at 5 °C	[101]
*Dicentrarchus labrax* burgers	Brewer’s spent grain extract	Supercritical CO_2_ extraction	0.69–1.77 mg GAE/g	N/A	5% (1:2 microencapsulation rate)	44.93–48.49% DPPH inhibition	The microencapsulation of the extract masked its bitter taste, and a positive sensory evaluation was obtained. Moreover, this sample showed a better antioxidant activity compared to the control	[109]
*Thunnus* sp. burgers	Dry olive paste flour	N/A	6.15–6.55 mg GAE/g	Tyrosol, caffeic acid and *p*-coumaric acid	10% *w*/*w*	84.87–85.77% DPPH inhibition	The addition of the extract increased the antioxidant activity of the final product, but it provoked a deterioration of sensory quality	[110]
*Oreochromis niloticus* fillets	Commercial standard	N/A	N/A	Nerolidol	1.0 mL/Kg of feed	<7.5 μmol CHP/g of tissue (LPO levels) <2 U DCF/mg of protein (ROS levels)	Nanoencapsulation of nerolidol promotes fish health by promoting growth and reducing free radical production and lipid damage	[111]

### 5.3. Natural Antioxidants for Oil Food Matrices

Susceptibility to oxidation is a major challenge for most edible vegetable oils during storage, cooking and processing, as it leads to rancid odors and off-flavors, thus reducing their shelf-life and nutritional value. Some of the most important factors for maintaining their stability are the presence of unsaturated fatty acids and the amount and type of antioxidants; however, these antioxidants are susceptible to oxygen, heat and light degradation [112]. In fact, natural antioxidants present in plant oils such as tocopherols, tocotrienols, carotenoids, phenolic compounds or sterols are dramatically reduced in refined oils [113], where total phenolic content decreased by more than 80%, and its antioxidant capacity decreased by about 60% [114]. Oxidation of edible oils is also accelerated by high temperatures reached when frying, increasing the subsequent formation of oxidative products, which are assimilated by the fried food. For instance, α-tocopherol oxidation products present in heated oils lead to deterioration in food quality [115] and these degraded products are also considered to be harmful for human health [113]. In order to avoid all of these mentioned issues, synthetic antioxidants such as TBHQ, BHA and BHT (Figure 1) have been demonstrated to be effective at fixing the oxidative stability of edible oils; however, they are being strongly questioned based on their possible negative effects on health and there is a growing trend towards the use of natural antioxidants [116].

Many plant extracts have proven to be good options as natural antioxidants to enhance both oxidative and thermal stability when added to edible oils. Sunflower oil oxidative stability improves during 24-day accelerated storage at 65 °C by adding the essential oil extracted from *Coriandrum sativum* L., which is rich in phenolic compounds, its efficiency being similar to TBHQ [117]. The term ‘accelerated’ is widely used in oxidative stability testing of food matrices such as oils and refers to an increase in oxidation due to an enhancement of the oxidation rate by temperature and not to additional oxidative reactions to those that may be observed at room temperature [41]. Similarly, the degradation resistance of sunflower oil during 30 days of storage at room temperature and under frying conditions at 180 °C was also demonstrated with the supplementation of this oil with *Pterospartum tridentatum* L. flower extract. In another study, purslane (*Portulaca oleracea* L.) leaf extract, rich in phenolic compounds and β-carotene (Figure 2), showed thermal stability similar to TBHQ in heated soybean oil at a concentration of 1500 ppm (Table 3) [118]. A recent study showed that the addition of a carotenoid-rich nutraceutical extract from *Lycium barbarum* L. can help to improve the oxidative stability when extra-virgin olive oil is subjected to a frying process [119]. Olive oil oxidative stability in accelerated storage conditions has also been improved when enriched with olive tree (*Olea europaea* L.) leaf extract due to its increase in polyphenol content, mainly rutin, luteolin-7-*O*-glucoside and quercitrin (Figure 4), which contribute to the radical scavenging activity [120]. The antioxidant activity of different plant extracts on the oxidative stability of pecan oil during storage was also studied, and it was concluded that caffeic acid (Figure 5) showed the strongest effect, demonstrating a higher effect than BHT and BHA and close to that of TBHQ [121]. 

In the case of omega-3-rich fish oils, a protective effect is observed when myricetin is added [122]. Dihydroquercetin (Figure 4) has also been successfully used to inhibit salmon oil oxidation, allowing up to eleven days of storage [123]. Sour orange (*Citrus aurantium* L.), bergamot (*Citrus bergamia* Risso and Poit) and grapefruit (*Citrus paradise* Macfad.) extracts were tested in order to improve fish lipid stability, with the sour orange albedo (peel inner layer) extracts being the most effective at reducing lipid oxidation. Moreover, unpleasant odors were also neutralized due to citrus composition [124].

The *Lamiaceae* family is well known for its antioxidant activity due to its content of phenolic compounds. *Teucrium polium* L. essential oil, which is rich in mono and sesquiterpene compounds, was studied as an antioxidant for canola oil and displayed higher protective effects against the oxidation of this oil in storage than BHA [125]. It has been shown that the use of a rosemary powder filtrate could be an effective way to protect rapeseed oil from oxidation during storage or cooking [126]. Rosemary and thyme (*Thymus capitatus* L.) extracts have also been proven to prevent oxidation in soybean oil during continuous heating for 24 h at 180 °C, preserving the polyphenol content as well. In the case of thyme, the antioxidant activity of its extract is related to its richness in thymol and carvacrol (Figure 2) [127]. In another study, the oxidative stability of heated soybean and sunflower oils enriched with herbal plant extracts showed that oregano (*Origanum vulgare* L.) extract provides greater protection of sunflower oil samples, while thyme extract is more effective in soybean oil when compared with the same oil without the addition of the herbal plant extracts or with the addition of the synthetic antioxidant BHA [128]. 

Food byproducts can also be an interesting alternative as sources of phenolic natural bioactive compounds with potential application in preventing oxidation of edible oils. Mango peel, a byproduct of industrial mango processing, is rich in phytochemical compounds. On the other hand, byproducts from the processing of pomegranate fruits represent more than 50% of its total weight. In this context, a recent study showed how a carotenoid extract from mango (*Mangifera indica*) peel is able to act as a natural antioxidant by protecting sunflower oil against lipid oxidation [129]. Pomegranate (*Punica granatum* L.) and orange (*Citrus sinensis* L.) peel extracts, both rich in phenolic compounds, showed strong protective effects against the oxidation of sunflower and soybean oils during storage at 65 °C [130]. Enrichment of refined sunflower, soybean and corn oils with pomegranate peel methanolic extract showed stronger antioxidant activity, thermal resistance, oxidative stability and shelf-life during accelerated storage than those treated with TBHQ, with the highest detected flavonoids being hesperidin and quercitrin (Figure 4) [131]. Another example of the advantageous use of waste materials generated by the food processing industry is the use of *Stevia rebaudiana* Bertoni stem waste against fish oil oxidation [132].

There is an increasing interest in exploring the use of encapsulation techniques for protecting and increasing the bioactive properties of natural antioxidant compounds added to edible oils. The use of microemulsions has been shown to greatly enhance the effect of anthocyanin rich blueberry (*Vaccinium corymbosum* L.) phenolic extracts on the prolongation of the oxidative stability of extra virgin olive oil when compared with the effect of native blueberry phenolic extracts [133]. Olive leaves are one of the richest sources of phenolic compounds among the different parts of the olive tree, oleuropein and its derivatives, such as hydroxytyrosol and tyrosol (Figure 5), being the most abundant. The antioxidant activity of olive leave extract encapsulated by nanoemulsions was evaluated in soybean oil, and it was concluded that it could be used to protect the properties of oil in storage in a similar way to TBHQ, though it is not useful for maintaining thermal stability [134]. The reduced release rate achieved with nanoencapsulation has also been shown to be effective in maintaining the antioxidant properties of *Hyssopus officinalis* L. extract in soybean oil (storage for 40 days at 60 °C), hence increasing the shelf-life of this oil. This extract is rich in phenolic compounds such as chlorogenic acid, rutin, quercetin and rosmarinic acid (Figure 5) [135].

**Table 3:** Effect of natural antioxidants on lipid stability of oil food matrices. MDA: malondialdehyde; N/A: not available; GAE: gallic acid equivalent; meq: milliequivalents; TPC: total phenolic content.

Product Used	Natural Source and Plant Organ	Extraction Method	TPC	Natural Bioactives Content	Substrate Concentration	Lipid Oxidation	Main Results	Reference
Sunflower oil	*Coriandrum* *sativum* L. stems and leaves essential oil	Hydrodistillation	N/A	Linalool (37.12% *w*/*w*), geranyl acetate (35.72% *w*/*w*) and menthol (5.07% *w*/*w*)	1200 ppm	60.1 meq O_2_/kg 0.20 μg MDA/mL	The addition of the essential oil at 1200 ppm increased the oxidative stability of sunflower oils and exerted a synergistic effect with TBHQ during accelerated storage of 24 days at 65 °C	[117]
Soybean oil	*Portulaca oleracea* L. leaves extract	Solvent extraction (hydroalcoholic)	151.7 mg GAE/g	N/A	1500 ppm	1.67 meq O_2_/kg	The extract improved thermal stability of soybean oil during heating (173 °C for 24 h) in a similar manner to TBHQ (100 ppm)	[118]
Extra-virgin olive oil	*Lycium barbarum* L. Goji berries extract	Solvent extraction (hydromethanolic)	N/A	Carotenoids (zeaxanthin dipalmitate)	1.5 mg/100 g oil	2 meq O_2_/kg	The decrease in the total phenolic content of the oil was lower when the carotenoid extract was added, and the mono- and polyunsaturated fatty acids remained mainly constant after 180 min frying at 180 °C	[119]
Olive oil	*Olea europaea* L. var. *sylvestris* powdered leaves	N/A	N/A	Luteolin-7-*O*-glucoside, rutin and quercetin-3-*O*-rhamnoside	10 g powdered oleaster leaves/100 mL olive oil	21.74 meq O_2_/kg	The enriched olive oil was more endurable to oxidation under storage in the dark at a temperature of 65 °C in closed glass bottles for 24 days	[120]
Pecan oil	Caffeic acid from commercial source	N/A	N/A	Caffeic acid	200 ppm	8 meq O_2_/kg <25 mg MDA/g	Caffeic acid inhibited oxidation of pecan oil effectively and it was stronger than BHT and BHA and close to TBHQ under storage in dark conditions at 60 °C for 20 days	[121]
Fish oil	*Citrus aurantium* L., *Citrus bergamia* Risso and Poit. and *Citrus paradisi* Macfad. fruit extracts	Solvent extraction (hydroalcoholic)	5.29, 1.31 and 4.86 g GAE/ 100 g	N/A	1000 mg/kg	5.21 mg MDA/kg	*Citrus aurantium* albedo extract had the best results in stabilization of lipid oxidation compared to the others during 5 weeks of storage at 25 °C	[124]
Canola oil	*Teucrium polium* L. fresh aerial parts extract	Hydrodistillation	N/A	Mono and sesquiterpenes	600 ppm	1.02 meq O_2_/kg	The essential oil showed higher antioxidant activity in canola oil compared with BHA 200 ppm after 60 days of storage at 25 °C	[125]
Rapeseed oil	*Salvia rosmarinus* L. (commercial grounded)	N/A	N/A	Phenolic diterpenes (carnosol, carnosic acid)	1.0–2.0% (*w*/*w*)	<20 nmol MDA/g	The use of ground rosemary powder could be an effective way to protect rapeseed oil from oxidation after 1.5 and 3 h of exposure to accelerated oxidation (120 °C)	[126]
Soybean oil	*Thymus capitatus* L. and *Salvia rosmarinus* L. dry leaves	N/A	N/A	Carnosol and carnosic acid in rosemary and thymol and carvacrol in thyme	6% (*w*/*w*)	N/A	The oil flavored with both extracts separately when continuous heating for 24 h at 180 °C have proved to avoid oxidation in soybean oil by preserving the polyphenol content	[127]
Soybean and sunflower oils	*Origanum majorana* L., *Thymus vulgaris* L. and *Origanum vulgare* L. leaves extract	Solvent extraction (hydroalcoholic)	N/A	Rosmarinic and caffeic acids	0.07%	179.41 t_ON_/°C (sunflower oil enriched with oregano extract) 182.13 t_ON_/°C (soybean oil enriched with thyme extract)	Oregano extract at concentration of 0.07% was effective for protection of sunflower oil against oxidation and thyme extract at concentration 0.07% improved the oxidative stability of soybean oil when compared with the same unenriched oil or with the addition of BHA (0.01%)	[128]
Sunflower and soybean oil	*Punica granatum* L. and *Citrus sinensis* L. fruit peel extracts	Solvent extraction (hydroalcoholic)	N/A	N/A	1200 ppm (*w*/*w*)	62.69–86.39 meq O_2_/kg (sunflower oil) 46.71–69.09 meq O_2_/kg (soybean oil)	A higher antioxidant activity of pomegranate and orange peel extracts compared to BHT (200 ppm) was observed after 24 days of storage at 65 °C	[130]
Sunflower, soybean and corn oils	*Punica granatum* L. fruit peel extract	Solvent extraction (petroleum ether, ethyl acetate, ethanol, methanol, water)	N/A	Hesperidine and quercetrin	200, 400 and 600 ppm	24-18-16 meq O_2_/kg (sunflower oil) 12-10-9 meq O_2_/kg (soybean oil) 5-4-3 meq O_2_/kg (corn oil)	Pomegranate peel extract exhibited stronger antioxidant activity than unenriched oils and TBHQ (200 ppm) during accelerated storage at 70 °C for 10 days	[131]
Fish oil	*Stevia rebaudiana* Bertoni stem extract	Aqueous extract	46.14 mg GAE/g	Vanillic acid 4-*O*-β-D-glucopyranoside, protocatechuic acid, caffeic acid, chlorogenic acid and cryptochlorogenic acid	1000 ppm	<500 meq O_2_/kg <130 mg MDA/kg	The stem water extract had significantly higher antioxidant activity against fish oil oxidation than the control (without extract) under 5 days of storage at 50 °C	[132]
Extra virgin olive oil	*Vaccinium corymbosum* L. extracts from fruits	Solvent extraction (hydroalcoholic)	276–417 mg GAE/100 g	Anthocyanins	0.2 g/3 g (phenolic extracts) 0.4 g/3 g (microemulsions of phenolic extracts, 2:1 ratio) 0.4 g/3 g (liposomes of phenolic extracts, 2:1 ratio)	N/A	The oxidative stability of extra virgin olive oil enriched with encapsulated blueberry phenolic extracts was significantly higher when compared with control (without extract) at the temperature of 120 °C. Moreover, phenolic extracts encapsulated in microemulsions had a stronger effect on the prolongation of olive oil oxidative stability in comparison with the extracts encapsulated in liposomes	[133]
Soybean oil	*Olea europaea* L. leaf extract	Solvent extraction (methanol)	206.81 mg GAE/g	Oleuropein, hydroxytyrosol and tyrosol	100, 200, 300 mg/kg	<20 meq O_2_/kg	The nanoencapsulated methanolic extract could control oxidation better than the unencapsulated extract and its oxidation protection was comparable with TBHQ (100–200 mg/kg) after 20 days of storage at 60 °C	[134]
Soybean oil	*Hyssopus officinalis* L. leaves extract	Solvent extraction (hydroalcoholic)	117.43 mg GAE/100 g	N/A	100, 200, 300, 400 ppm	2 meq O_2_/kg	Nanocapsules carrying natural phenolic extracts increased the antioxidant activity of the oil compared with TBHQ (100 ppm) after 40 days of storage at 60 °C	[135]

### 5.4. Natural Antioxidants for Vegetables and Juice Food Matrices 

Industrial processing of fruits and vegetables includes, in some cases, peeling, cutting, blending or generation of flours, and canning, dehydrating or elaboration of juices or jellies. All of these transformations increase the oxidation risk in these food matrices, as they are in closer contact with atmospheric oxygen and other prooxidant compounds such as metals [136]. 

In order to prevent the oxidation of these processed plant food matrices, natural alternatives have been pursued in recent years, such as monoterpenes from essential oils like oregano (*Origanum vulgare*), which exhibit a high antioxidant activity as described above. For example, carvacrol and thymol (Figure 2) showed good preservation of sensory and antioxidant properties in roasted sunflower seeds, while sabinene hydrate (Figure 2) exhibited better behavior preventing peroxide formation in these same seeds [137]. Carvacrol is also able to reduce fruit decay in berries [138] and thymol and other terpenoids, such as menthol (Figure 2), are able to maintain better fruit quality in strawberries and tomatoes and maintain increased levels of sugars, organic acids, anthocyanins and antioxidant capacity in these fruits [139,140]. However, the sensory acceptability of cooked, canned vegetables treated with carvacrol and thymol as antioxidants has a negative impact due to their strong flavors, an effect that can be prevented by mixing these phytochemicals with food-grade polymers such as cyclodextrins [141,142]. 

Plant polyphenols can also be used in shelf-life extension and quality maintenance of certain fruits, such as litchi (*Litchi chinensis*), which suffers from the accumulation of ROS species and an enhanced polyphenol oxidase activity in its pericarp, mainly in its anthocyanidins, causing browning after its harvest [143]. Tea polyphenols are able to inhibit or retard this browning process during litchi storage, inhibiting the polyphenol oxidase enzyme and reducing lipid peroxidation (a key factor for rancid odor) [144]. Another plant secondary metabolite that inhibits the polyphenol oxidase enzyme is salicylic acid (Figure 2), which is able to delay browning in the Chinese chestnut (*Castanea mollisima*) [145]. In a similar way, the browning process in mashed potatoes and apples (also due to polyphenol oxidase activity) can be inhibited by the phenolic fraction of rice bran extract, where *p*-coumaric acid (Figure 5) is the active antioxidant, or by the addition of the amino acid L-cysteine (Figure 2) [146,147].

Polyphenols, including their aglycons, usually present a bitter and/or astringent taste, a factor that must be taken into account when using these plant antioxidants in some food matrices, such as juices or canned fruit [148]. Some examples are astringent proanthocyanidins (such as catechin oligomers) from grape skin [149], bitter naringenin or hesperetin (Figure 4) glycosides from citrus fruits or bitter isoflavones (such as genistein or daidzein) in soy products (Figure 4) [148]. As a way to solve these unpleasant taste problems, diverse masking technologies can be used to improve consumer acceptance of processed foods containing these plant antioxidants, such as nanocapsules or nanoemulsions [150,151,152,153]. In the specific case of juices, solubility of the added plant antioxidants is a key factor, which is higher in the case of flavonoid glycosides [154]. 

## 6. Packaging Strategies Using Natural Antioxidants

Antimicrobial and antioxidant packaging appears to be one of the most promising applications of active food packaging technology [155]. In fact, the demand for antioxidant active packaging is increasing due to its major consumer acceptance in comparison to chemical antioxidants [156]. Focusing on antioxidant packaging, the two main approaches are based on the release of antioxidants to the food and the scavenging of undesirable compounds, such as oxygen or radical oxidative species [157].

One of the ways to improve food quality is to incorporate phenolic compounds such as curcumin (Figure 2), quercetin and catechin (Figure 4) into the foil matrix. It has been shown that the use of phenolic compounds in packaging foil extends product shelf-life and has good antioxidant properties, preventing lipid oxidation in fresh ground pork [158]. Quercetin has also been used to prepare a multifunctional antioxidant film mixing it with polyvinyl alcohol (PVA). This resulting film was called quercetin aggregation-induced emission (AIE) composite film and it was used to extend the shelf-life of bananas and apples. It also shows a sensitive AIE enhancement, which occurs when the film is exposed to foods containing Al^3+^ residues and to seafood containing biogenic amines, produced during spoilage. Thus, this film has the potential to be used as a smart and green packaging material, sensing food quality fluorescently and extending food storage times [159]. Another study developed functional films by solution casting using three different types of carbohydrates (agar, chitosan and carrageenan) and curcumin. These films exhibited strong antioxidant activity and some antibacterial activity [160]. In another experiment, chitosan and curcumin were combined with cellulose, forming a biodegradable and active film. This strategy displayed the best antioxidant activity in a fatty food system, suggesting it could be a good active packaging material for foods with high fat content [161].

Antioxidant active materials have also been developed with films based on tea extracts, with properties showing good antioxidant activity and low migration rates from films to food [162]. In this fashion, a new antioxidant polyamide was prepared by total immersion in active green tea extract for 48 h, so antioxidants were adsorbed onto the surface. This film showed excellent antioxidant properties in fresh minced meat refrigerated for 23 days [163]. Green tea extract can also be encapsulated for the preparation of active materials. The effectiveness of the developed packaging was checked with in vivo experiments and the extension of shelf-life of fresh minced pork meat was successfully achieved [164].

There are other non-phenolic antioxidant compounds used in food packaging, like caffeine (Figure 2) and carotenoids (Figure 2). Carotenoids have been applied not only directly into food, but also for active packaging aiming to increase the protection from light or oxygen permeability. As well, carotenoids help strengthen the packaging polymers during storage and processing due to their antioxidant activity and capacity to absorb UV light. A recent study described that poly-(lactic acid) films with carotenoids (bixin, ß-carotene and lycopene) (Figure 2) can be useful for sunflower oil preservation [165]. Another example of active packaging films is based on starch/polyvinyl alcohol incorporated with betalains-rich red pitaya (*Hylocereus* polyrhizus (F.A.C. Weber) Britton and Rose) peel extract. The incorporation of this extract improved the mechanical, antioxidant and antimicrobial properties of the films and its protective capacity against UV radiation [166]. 

Apart from the evident advantages of using active food packaging, biodegradable coatings can also be useful in reducing the high amount of plastic waste [167]. Recently, new biodegradable chitosan-gelatin based films containing quercetin-starch have been developed. It was observed that the film containing quercetin-starch increased the antioxidant activity in comparison to the control without the flavonoid incorporated [168]. Biodegradable poly-(ester-urethane) film, based on a triblock copolymer of poly-(lactic acid) and poly-(ε-caprolactone) and further loaded with catechin (Figure 4) as an antioxidant agent, was developed. This new material showed good catechin release and effective antioxidant activity and appropriate disintegration in compost [169].

Finally, as far as macroalgae are concerned, *Fucus vesiculosus* L. is a brown edible seaweed and its hydroethanolic extracts were incorporated into a whey protein film to generate an active packaging capable of controlling lipid oxidation in chicken breasts during a total storage time of 25 days [170]. 

## 7. Conclusions

Current industrial food processes have a broad array of techniques and chemical synthesis additives available as antioxidants for their use in food preservation. Some of these additives are used to prolong shelf-life (mainly to avoid lipid oxidation and aesthetic deterioration). However, some of these techniques or additives may generate health concerns among consumers. Scientific data, together with an increasing consumer perception of the need for safer and more natural food processing techniques and additives in recent years has resulted in increased efforts at scientific and industrial levels to be devoted to the use of plant metabolites, such as polyphenols and terpenoids, among others, as food antioxidants. The antioxidant activities for some of these plant nutraceuticals have been tested in vitro and in the stored food matrix, shedding natural light on the complex field of food additives and preservatives. Together with their important antioxidant activities, some of these plant secondary metabolites possess other interesting properties also, such as being cardioprotective (such as astaxanthin), an antitumor (such as curcumin) or bactericidal (such as quercetin), which adds value to their use in some food matrixes pursuing their marketing as functional foods. Additionally, the national legislations may be different for a given antioxidant, as in the case of astaxanthin, which is registered in US law as a color agent, but not permitted in the EU.
